# Topography of Photochemical Initiation in Molecular Materials

**DOI:** 10.3390/molecules181114148

**Published:** 2013-11-15

**Authors:** Edward D. Aluker, Alexander G. Krechetov, Anatoly Y. Mitrofanov, Anton S. Zverev, Maija M. Kuklja

**Affiliations:** 1 Department of Physical Chemistry, Kemerovo State University, Kemerovo 650043, Russia; 2 Department of Materials Science and Engineering, University of Maryland, College Park, MD 20742, USA

**Keywords:** molecular energetic materials, high explosive decomposition, laser excitation, PETN, excitonic mechanism of initiation

## Abstract

We propose a fluctuation model of the photochemical initiation of an explosive chain reaction in energetic materials. In accordance with the developed model, density fluctuations of photo-excited molecules serve as reaction nucleation sites due to the stochastic character of interactions between photons and energetic molecules. A further development of the reaction is determined by a competition of two processes. The first process is growth in size of the isolated reaction cell, leading to a micro-explosion and release of the material from the cell towards the sample surface. The second process is the overlap of reaction cells due to an increase in their size, leading to the formation of a continuous reaction zone and culminating in a macro-explosion, *i.e.*, explosion of the entire area, covering a large part of the volume of the sample. Within the proposed analytical model, we derived expressions of the explosion probability and the duration of the induction period as a function of the initiation energy (exposure). An experimental verification of the model was performed by exploring the initiation of pentaerythritol tetranitrate (PETN) with the first harmonic of YAG: Nd laser excitation (1,064 nm, 10 ns), which has confirmed the adequacy of the model. This validation allowed us to make a few quantitative assessments and predictions. For example, there must be a few dozen optically excited molecules produced by the initial fluctuations for the explosive decomposition reaction to occur and the life-time of an isolated cell before the micro-explosion must be of the order of microseconds.

## 1. Introduction

Laser initiation of high energy density materials has long attracted the attention of researchers [1,2,3,4,5,6,7,8] because of exciting new opportunities in fundamental science and applied technology for various applications, especially those relevant to improving safety of producing and handling high explosive materials and devices [9,10], sensors [11,12,13] and detectors [14,15,16]. A mature, popular opinion shared by many researchers is that the mechanism of laser initiation is of a thermal nature, and the laser light is used as a source of heat for absorbing small inclusions (e.g., soot particles), which act as “hot spots” to originate the thermal explosive decomposition reaction [2,4,9,10]. A critical analysis of a large body of literature however leads to a worrying conclusion that the thermochemical mechanism of the initiation process, is, in fact, simply postulated, without any consideration of possible alternative mechanisms [5,6].

Contrary to the popular opinion, there is both theoretical and experimental evidence that electronically excited states play a crucial role in triggering decomposition chemistry in molecular materials. For example, quantum-chemical calculations of gas-phase decomposition of 1,1-diamino-2,2-dinitroethylene (DADNE) molecules (C_2_H_4_N_4_O_4_, also known as FOX-7) [17] established that charging and excitation can not only reduce the activation barriers for decomposition reactions, but also change the dominating chemistry from endo- to exothermic type. This research provided strong support to an excitonic mechanism of initiation of chemistry that was theoretically proposed earlier [18,19]. Experimentally, it was demonstrated that the laser initiation can be photochemical in nature at least for some systems, such as heavy metal azides and secondary explosives, cyclotrimethylene trinitramine (RDX, C_3_H_6_N_6_O_6_) [20], pentaerythritol tetranitrate (PETN, C_5_H_8_N_4_O_12_) [5,21,22,23], and furazan-based molecules [24,25]. In particular, it was observed that the (non-thermal) photoexcitation of PETN molecules does lead to the formation of reactive radicals, and that the further explosive decomposition reaction proceeds by a chain or autocatalytic (chain explosion) mechanism [5,21,22]. Recent quantum-chemical calculations of thermal decomposition of PETN molecules, crystals, and crystal surfaces [26] ruled out a possibility of thermal initiation of PETN observed in those experiments [5,21,22]. The first principles analysis of the ground state reaction mechanisms convincingly demonstrated that the lowest theoretically obtained activation barriers (~35–36 kcal/mol) [26] as well as the experimental range (32.6 to 47.3 kcal/mol) [27,28,29,30,31] were found to be inconsistent with the experimental energy of laser initiation (1.17 eV = 27.0 kcal/mol) [5,21,22]. Thus, the drawn conclusions lend some additional support to the notion that an excited state of a PETN molecule, created by the absorption of a photon, rather than by a thermal energy, was the primary act of initiation in laser irradiation experiments [5,22,32].

Due to its stochastic nature, this process is inevitably accompanied by fluctuations that result in the emergence of microscopic regions with high density of excited molecules. A similar effect is well known in radiation chemistry, where cells with high ionization density are formed in the tracks of nuclear particles due to the stochastic nature of the ionization process [33]. Similarly, in the photo-induced decomposition, these micro-regions with a high concentration of excited molecules can act as nucleation reaction cells, which effectively trigger a chain reaction of explosive decomposition. The presence of such reactive cells observed experimentally [34,35,36] is consistent with findings that the chemical activity of energetic materials originated in specific localized spots rather than uniformly across the sample [20].

It was established that the pulsed laser and electron beam initiation of silver azide results in the occurrence of pre-detonation luminescence, accompanying the initial stage of the explosive decomposition [34,35,36]. It was also shown that the chemical reaction is born at discrete sites, which act as nucleation reaction micro-cells in the not-yet fractured sample (the pre-explosion stage). Further growth of the linear dimensions of micro-cells occurs at a rate of the order of 10^3^ m/s, which causes their overlap in a time of the order of 10^−7^ s and leads to the explosion [34,35,36]. We emphasize that the generation of reactive micro-cells, considered here, is determined by the stochastic nature of the initiation process; and neither the presence of structural defects nor the thermal lattice fluctuations that are capable of trapping electrons and holes produced by the optical excitation [37] are implicitly included in our consideration at this point.

Despite existing experimental data and vast interest in gaining a better understanding of ultra-fast and chain processes in materials, fundamentals of the chain reaction of explosive decomposition remained practically unexplored in the field of laser initiation of detonation. The role of density fluctuations of photo-excited molecules in triggering the chemical reactions in energetic materials is yet to be established. In this study, aimed at making the first step towards the development of the phenomenological model of photochemical initiation in explosives, we analyze the process of photo-initiation of chemical decomposition of energetic materials from a point of view of stochastic interactions between photons and energetic molecules. We suggest an analytical fluctuation model, in which local splashes of molecular photo-excitation density serve as reaction nucleation sites, and the reaction propagation depends on the interplay of increasing size of these sites and their overlap. Further, we validate the model with experiments on the initiation of PETN with the first harmonic of YAG: Nd laser excitation (1,064 nm, 10 ns) and check our predictions. Although we generally follow the ignition and growth concept in our study, there are significantly different aspects in our model in comparison to the widely used Lee-Tarver model, which describes the shock initiation of heterogeneous solid explosives by Lagrangian hydrodynamic code [38,39]. In the Lee-Tarver ignition and growth model, the leading shock wave of an initiating pulse is assumed to thermally ignite a small fraction of the explosive at localized heated regions. Characterized by the time resolution of microseconds and space resolution of microns [40], the model uses a set of fitted parameters to simulate how these ignited regions grow (or fail to grow) as material is consumed at their boundaries to lead to a detonation (or a failure of detonation). Our model is dealing with much faster process of photo-initiation that triggers chemistry at a femtosecond time scale and hence is not described within the classical mesoscale Lee-Tarver model.

## 2. Theoretical Model

Three well-established provisions laid the groundwork for the proposed model. First, the stochastic nature of the interaction of photons with the energetic molecules at the photochemical initiation leads to fluctuations of the density of the excited molecules. In the case of fairly large fluctuations, conditions for the emergence of a self-sustaining chain reaction can be satisfied; in other words, those fluctuations are beginning to act as nucleation of microcells of the explosive decomposition reaction. With the reaction progressing across the sample, the sizes of these reaction cells are growing, leading to their overlap, which forms the explosion zone covering the whole sample or, at least, a significant part of it. An increase of the initiating radiation intensity leads to an increase in the concentration of reaction cells and consequently reduces the time required for their overlap. The growth rate of the linear sizes of the cells does not depend on the concentration (it is ~1 km/s for silver azide [34,35] and 0.9 km/s was measured for PETN [41]).

Second, at the photo-excitation near the initiation threshold, the surface of unexploded samples exhibit cavities and pits [9,10], *i.e.*, there is a local ejection of matter from the reaction cell region. This “early” material release from the cell region effectively stops the progress of the reaction and therefore may serve as a suppressing factor [42].

Third, confined samples (samples encapsulated in a closed metal shell) exhibit a lower initiation threshold [4,9,10] than open samples of the same material. Speculating, we suggest that the most likely reason for this is that the presence of the shell precludes the “early” material release from the reaction cells, thus the confinement serves to minimize the energy losses.

Within this context, it seems that a reasonable, well-grounded scenario of the photochemical initiation of explosive decomposition is the following. The chain reaction begins in the micro-regions of the fluctuation of increasing density of the excited molecules. At low concentrations of these regions, corresponding to low exposures of initiation, any significant overlap of these cells does not have sufficient time to occur prior to the ejection of matter from the reaction cells (a micro-explosion cell), and the reaction dies out in the formation of cavities and/or pits. At high concentrations of cells, their overlap occurs before the micro-explosion of an individual cell, and we observe an explosion of a significant part of the sample (*i.e.*, macro-explosion).

Thus, the probability of explosion is determined by the ratio of two times, the average lifetime of an isolated reaction cell, *i.e.*, a period from cell birth to its micro-explosion, τ_0_, and the average time of cells overlapping, τ*_e_*, which is a function of their concentration, and, consequently, the initiation intensity.

We now turn to a quantitative analysis of the situation. Assuming that *k* excited molecules are required to initiate the decomposition reaction in a cell with a linear size *b_0_* and a volume *b_0_^3^*, the number of cells that contain a total of *k* excitations is *m* *=* *k/(Nb_0_^3^)*, where *N* is an average concentration of excited molecules generated by the laser irradiation. The number of possible distributions of *k* molecules over *m* cells is *m^k^*. The probability of finding all *k* excited molecules in the same cell (or in the volume *b_0_^3^*) is *p* = *m^1^*^−*k*^ = (*Nb*_0_^3^/*k*)*^k^*^−1^. The concentration of such cells that contain *k* excited molecules, *n* = *p*/*b*_0_^3^ will be: 
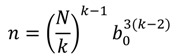
(1)

At low absorption, the *N* value is proportional to initiation exposure *H* (*i.e.*, the energy of the initiating light pulse over a surface area unit) with η as a coefficient:

*N* = η*H* (2)

By combining Equations (1) and (2), one obtains:

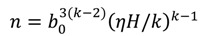
(3)

Each of these cells originates the chain reaction of explosive decomposition, and the size of each cell grows as the reaction progresses [34,35,36].

Next, we estimate the time required for individual cells to overlap, τ_e_; this is also the time of the formation of the explosion, covering the entire sample. As a simple estimate of the τ_e_ we take the time needed for the reaction front to propagate at a distance equal to the average distance between cells, *n^1/3^*, 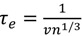, where *υ* is a velocity of the reaction front (or the growth rate of the linear dimensions of the reaction cell). Then, taking into account Equation (3), we derive an estimate of *τ_e_*:

*τ_e_* = *τ*_0_ [*C*/*H*]^(k−1)/3^ (4)

where:


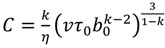
.

Equation (4) is deliberately multiplied and divided by *τ_0_* for mathematical convenience. An additional benefit here is that this way brings a clear physical meaning to the constant C, which can be attributed to the initiation exposition, corresponding to the explosion probability of 50%.

In the case of a chain character of the process (and this is the case discussed in the article), the propagation of the reaction zone front is defined by a diffusion of active radicals [43]. In our case, the concentration of these radicals is created due to the absorption of photons by PETN molecules, and the increase in their concentration is the result of a chain process in which they participate. For the time being, details of the process, the structure and properties of the active radicals remain to be established. In case these active radicals are electronic excitations of the crystal lattice (electrons, holes, excitons), the velocity of the thermal diffusion of the particles, which determines the reaction rate, is of the order of 10^5^ cm/s (ref. [3,34,35,36]). Hence we take this amount as an estimate of the growth rate of the linear dimensions of the micro reaction centers. We emphasize that a change of this magnitude does not alter the qualitative picture of the phenomenon, but rather affects only quantitative characteristics.

The probability of explosion of the sample, *W*, will be determined by the relative rates of two competing processes, the rate of overlapping of cells, 1/τ_e_, resulting in the formation of the explosion zone and, consequently, in macro-explosion, and the speed of micro-explosions in individual cells, *1/τ_0_*, leading only to the formation of cavities as a result of the ejection of material outside the cell, *i.e.*, suppressed process. Statistically, in such a two-channel system [44]:

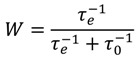
(5)

From Equations (4) and (5), we derive the probability of explosion as:

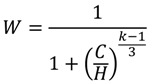
(6)

## 3. Experimental Validation of the Model

The adequacy of the proposed model can be tested in a relatively simple and effective way. We will describe the experimental data on the probability of explosion as a function of initiation exposure, *W*, and the delay time with respect to the initiating impulse, τ_e_ (that is the duration of the induction period) by using expressions Equations (4) and (6).

**Figure 1:**
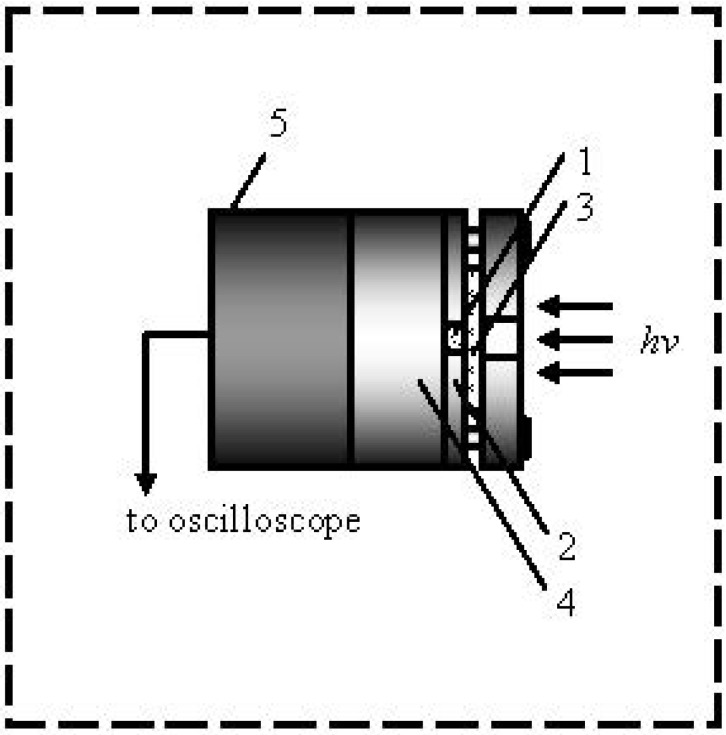
The components of the explosion chamber, (1) sample, (2) shell, (3) glass plate, (4) steel substrate, and (5) acoustic sensor, are shown.

The pentaerythritol tetranitrate (PETN, C_5_H_8_N_4_O_12_) samples for our experiments were synthesized by the nitration of pentaerythritol with concentrated nitric acid. They were purified by recrystallization from acetone and repeated recrystallization in hexane to obtain a powder of PETN with a grain size of 100 microns. The samples under study consisted of tablets having a diameter of 3 mm and a thickness of 0.5 mm, pressed into a stainless steel shell (Figure 1) at a pressure of 200 MPa. The density of the samples was 1.7 g/cm^3^ (density of a PETN single crystal is 1.77 g/cm^3^). A schematic representation of the explosion chamber is shown in Figure 1.

Initiation was carried out with 10 ns pulses of the first harmonic (1,064 nm) YAG: Nd laser LDPL10M. Initiation exposure was determined using the pyroelectric head PE50BF-DIF-C (Ophir Photonics, Jerusalem, Israel) and was constantly monitored by a signal of the calibrated photodiode. Special care was taken to ensure a fairly homogeneous distribution of the excitation intensity over the sample surface, and only the central part of the laser beam was used for initiation. The beam diameter is 6 mm and the tablets are 3 mm in diameter. The energy deviation of the initiating pulse did not exceed 3%.

**Figure 2:**
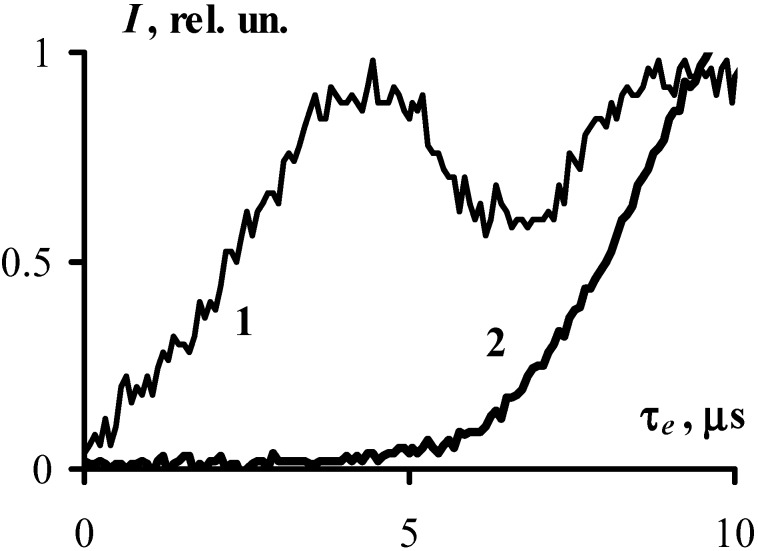
The oscillogram obtained from the acoustic sensor represents (1) the signal generated by the laser irradiation of the empty experimental cell and (2) the signal resulted from the sample explosion. The temporal resolution of the oscilloscope is 0.8 ns. The resolution of the acoustic sensor (~3 µs) is determined by its response to the laser pulse excitation. The temporal resolution of the apparatus primarily affects the translation of the pulse shape and to a much lesser degree the measurement precision of the delay between pulses. The use of this fairly rough acoustic sensor is prudent due to its high stability against explosive effects.

The acoustic sensor in the experimental set up enabled a reliable timing of the start of the explosive decomposition of the sample with respect to the initiating pulse as the acoustic sensor detects two pulses, the first pulse appears as the result of the impact of the initiating laser pulse adjacent to the back side of the sample surface of the explosion chamber, the second pulse is the result of the explosion of the sample. The time interval between the leading edges of the pulses (at the level of the amplitude of 0.1) was taken as the duration of the induction period, *τ_e_*. Examples of the obtained oscillograms are given in Figure 2.

**Figure 3:**
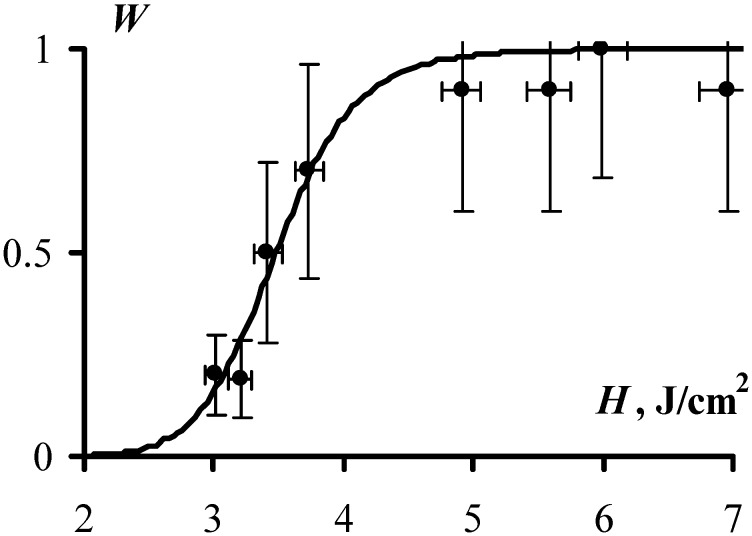
The probability of explosion as a function of initiation exposure (1064 nm, 10 ns) is obtained as an experimental result of initiation of at least ten PETN samples (dots) and as a result of approximating the obtained experimental data by Equation (6) with *C* = 3.5 ± 0.1 J/cm^2^ and *k* = 35 ± 8 (solid line).

**Figure 4:**
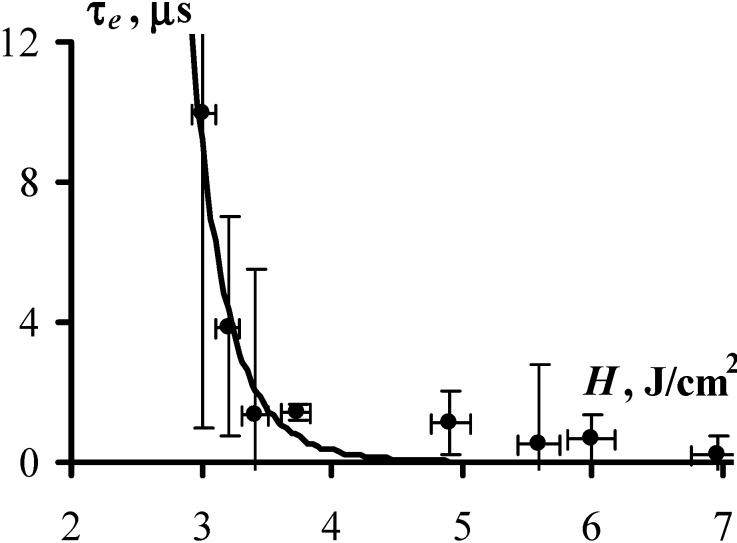
The duration of the induction period as a function of initiation exposure (1,064 nm, 10 ns) is demonstrated with the experimentally measured average time prior to explosion with at least ten PETN samples exploded at each *H* (dots) and with approximation by Equation (4) with *C* = 3.5 J/cm^2^, *k* = 35, and τ_0_ = 1.7 ± 0.3 µs (solid line). The parameters *C* and *k* are obtained from Figure 3. The values of τ_0_ were calculated from the slop of the τ_e_~f(1/W-1). The large error of the first point on the graph (at *H* = 3 J/cm^2^) results from a small number of successful measurements as only two samples exploded out of ten. Good correspondence between calculated and experimental data is observed despite the large uncertainty of the individual measurements.

Figure 3 and Figure 4 present the obtained experimental results, showing the dependence of the explosion probability, *W*, and the duration of the induction period, *τ_e_*, on exposure initiation. At a glance, the experimental data are well approximated by the expressions Equations (4) and (6), which provides a solid argument in favor of the adequacy of the proposed model. More specifically, the values of fitting parameters *k* and *τ_0_*, (which both have direct physical meaning) were obtained from the experimentally measured dependencies, *W(H)* and *τ_e_(H)* and turned out to be *k* = 35 and τ_0_ = 1.7 µs. The expressions Equations (4) and (6) yield the same values of *k* and τ_0_.

We note that both estimates, *k* = 35 excited molecules and τ_0_ = 1.7 µs, although appear quite reasonable, refer to the conditions of the experiment. Obviously, the reduction in strength of the metal shell surrounding the sample may lead to a decrease of τ_0_ and increase of *k*. Therefore the results obtained in this experiment and the values of *k* and τ_0_ are determined, generally speaking, only at the level of the order of magnitude in such a way that *k* is of the order of ten molecules and τ_0_ is of the order of a µs.

The value of *C*, which corresponds to the initiation exposure that provides 50% probability of explosion (see Equation (6)), is calculated as *C* = 3.5 J/cm^2^, also happens to be the same when approximated by both dependences (Figure 3 and Figure 4).

It is doubtful that such agreements of three different parameters *k*, τ_0_ and *C*, obtained from different observables can be achieved accidently.

We also emphasize that the present model allows us to consistently explain from a single point of view the facts that appeared earlier as completely unrelated, in particular, the fluctuation micro-scale origin of photochemical initiation [34,35,36], the formation of cavities on the surface of the unexploded samples at the excitation near the photo-initiation threshold [9,10], and the impact of the sample case on the initiation threshold [2,4,9,10].

## 4. Discussion and Conclusions

A fluctuation model of initiation of explosive chain decomposition is proposed. The model is based on the notion that interactions between the light of the laser excitation and molecules of an energetic material result in the photo-excitation density fluctuations in the sample; these fluctuations (micro-regions of the increased excitation density) serve as reaction nucleation sites. Unlike Lee-Tarver nucleation and growth model [38] in its statistical formalism [40], which assigns hotspot density (physical density fluctuations, or particles) to the nucleation sites, our model attributes density of electronic excitations (quasiparticles) to the nucleation sites. Two competing and intertwined processes define how the chain reaction will progress. The growth in size of the isolated reaction cell leads to a micro-explosion and injects material outside of the cell towards the surface. The overlap of reaction cells due to an increase in their size leads to the formation of a continuous reaction zone and ultimately to a macro-explosion. The developed analytical model describes the explosion probability and the duration of the induction period as a function of initiation energy. We then performed experiments on the YAG: Nd (1,064 nm, 10 ns) laser initiation of PETN. A comparison of the theoretical model and experimental data predicted that in order for laser excitation to initiate the explosive decomposition of an energetic material (not limited to PETN samples), at least a few dozen (~30–40) optically excited molecules must be locally (in the individual cell) affected by the initial density fluctuation and the life-time of the cell (before the micro-explosion) must be at least a few microseconds.

We suggest that several important arguments evidence for adequacy of the proposed model. Specifically: *(i)* the experimental data are well approximated by theoretically derived functions of the induction period, τ_e_, and the probability of explosion, *W*, depending on the initiation exposure, *H*, as illustrated in Figure 3 and Figure 4; *(ii)* the values of the constants that are included in the different dependencies, coincide, and the estimated micro parameters (*k*, τ_0_) yield reasonable values; and finally, *(iii)* the seemingly disparate facts: the fluctuation-triggered origin of the chain reaction, the formation of pits and cavities on the surface of the non-exploded samples, and the role of the sample’s confinement on the initiation threshold of high explosive materials, can be explained now from the common point of view.

We believe that this phenomenological model signifies a breakthrough that will serve as yet another critical building block towards design of a united microscopic theory of initiation of detonation. In accordance with the proposed model, the first step in the explosive decomposition reaction is attributed to the optical absorption of a photon by an individual molecule. This perturbation drives the system (the molecule) to the excited state and triggers a chain of processes. The absorption therefore is the earliest elementary act of a photochemical reaction. Contrary to this, the first step of the thermochemical initiation is the absorption of a group of photons by a “hot spot” (a defect, deformation, impurity, or other imperfection in the material), locally heating the material up to a critical temperature to trigger a thermal decomposition, which is not described by the developed model. As was demonstrated in this research, the concept of photo-excitation density fluctuations laid a foundation to design the experiments. The theoretical model based on the concept was able to accurately describe and interpret the performed measurement, which evidences in favor of photo- (rather than thermo-) activation of chemistry of the laser excitation explored here. Hence, the developed model provides a new and attractive tool to study photo-activated processes relevant to stability/decomposition and chain reactions in materials as it clearly discriminates actions and consequences of photo- and thermochemical mechanisms, which was not attainable before.

There are reasons that prompted us to limit our consideration in this article only to the photo-chemical mechanism, without involving other initiation scenarios, in particular the photo-thermal initiation mechanism of hot spots [5,21,22,32]. First, this limitation allows us to perform an experiment on photo-initiation of PETN, a well-studied energetic material, and to test the predictions of the proposed model. Second, it is reliably established in a series of articles [5,21,22,25,32] that the regime of photochemical initiation, *i.e.*, photo-excitation of PETN molecules, actually takes place. It is also shown that the photo-initiation leads to the formation of active radicals, providing the further degradation of the material by an explosive chain decomposition reaction, or an autocatalytic process. Thus, the offered conditions are *ideal* not only for the development of an appropriate model, but also for the experimental verification of its adequacy. Other mechanisms of photo-initiation, especially the most popular photo-thermal initiation mechanism, fall short in providing a similarly appealing situation and require a lot of preliminary work that goes far beyond the scope of this article.
